# Effect of a Bone Marrow-Derived Extracellular Matrix on Cell Adhesion and Neural Induction of Dental Pulp Stem Cells

**DOI:** 10.3389/fcell.2020.00100

**Published:** 2020-03-06

**Authors:** Samuele Laudani, Valentina La Cognata, Rosario Iemmolo, Gabriele Bonaventura, Giusy Villaggio, Salvatore Saccone, Maria Luisa Barcellona, Sebastiano Cavallaro, Fulvia Sinatra

**Affiliations:** ^1^Section of Biology and Genetic, Department of Biomedical and Biotechnological Sciences, University of Catania, Catania, Italy; ^2^Institute for Biomedical Research and Innovation, Italian National Research Council, Catania, Italy; ^3^Section of Animal Biology, Department of Biological, Geological and Environmental Sciences, University of Catania, Catania, Italy; ^4^Section of Biochemistry, Department of Pharmaceutical Sciences, University of Catania, Catania, Italy

**Keywords:** cell differentiation, dental pulp cells, ECM, neuronal-like stem cells, neuronal markers

## Abstract

Extracellular matrix (ECM) represents an essential component of the cellular niche. In this conditioned microenvironment, the proliferation rates and differentiation states of stem cells are regulated by several factors. In contrast, in *in vitro* experimental models, cell growth, or induction procedures toward specific cell lines usually occur in contact with plastic, glass, or biogel supports. In this study, we evaluated the effect of a decellularized ECM, derived from bone marrow stem cells, on the neuronal differentiation of mesenchymal stem cells (MSCs) extracted from dental pulp (Dental Pulp Stem Cells – DPSCs). Since DPSCs derive from neuroectodermal embryonic precursors, they are thought to have a greater propensity toward neuronal differentiation than MSCs isolated from other sources. We hypothesized that the presence of a decellularized ECM scaffold could act positively on neuronal-DPSC differentiation through reproduction of an *in vivo*-like microenvironment. Results from scanning electron microscopy, immunofluorescence, and gene expression assays showed that ECM is able to positively influence the morphology of cells and their distribution and the expression of specific neuronal markers (i.e., *NF-L*, *NF-M*, *NF-H*, *PAX6*, *MAP2*).

## Introduction

Adult mesenchymal stem cells (MSCs) are a population of multipotent cells characterized by self-renewal and differentiation potential under suitable stimuli. They are located in anatomical spaces named *cellular niche*s where paracrine factors, cell-to-cell contacts, and extracellular influences regulate their proliferation and differentiation (Scadden, 2006). MSCs were originally found in the bone marrow (BMMSC) of mouse and showed osteogenic, adipogenic, and chondrogenic properties (Tavassoli and Crosby, 1968; Friedenstein et al., 1970). They have also been isolated from other anatomical districts including adipose tissue, synovial liquid, skeletal muscle, lung, human umbilical cord, and oral cavity. In particular, the oral cavity hosts several cell populations expressing both MSC-like features and a particular ability to differentiate toward specific cell-lines, which depends primarily on their embryonal origin (Kern et al., 2006; Isobe et al., 2016). Dental pulp stem cells (DPSCs) and the recently discovered human periapical cyst MSCs (hPCy-MSCs), for example, have been found highly attractive by researchers for clinical purposes because of their simple protocol of isolation and multiple and efficient applications in bone, dental, and neural tissue regeneration (Tatullo et al., 2017; Bonaventura et al., 2019).

Dental pulp stem cells are known to uniformly express the cell−surface markers CD29, CD44, CD90, CD105, CD117, and CD146 (Dominici et al., 2006; Gervois et al., 2015) and, similarly to MSCs, they have the ability to differentiate into chondrocytic, adipogenic, and osteogenic cell types (Grottkau et al., 2010; Lee et al., 2011; Isobe et al., 2016; Yi et al., 2017). Moreover, because of their origin from neuronal crest, DPSCs are able to produce some neurotrophic factors (e.g., nerve growth factor – NGF or glial cell line-derived neurotrophic factor – GDNF) that have a neuroprotective action *in vitro* (Hoffer et al., 1994; Hou et al., 1996; Nosrat et al., 2001, 2004; Smith et al., 2015). Furthermore, DPSCs are able to differentiate, under opportune stimuli, into neuronal-like stem cells (NSC) expressing typical neuronal markers such as PAX6 (Zhang et al., 2010), microtubule-associated protein type 2 (MAP2) (Mohan and John, 2015), Vimentin (Wilkinson et al., 1990; Sun et al., 1997; Yabe et al., 2003; Dubey et al., 2004; Vinci et al., 2016), and neurofilaments (NF) (Gentil et al., 2015; Lowery et al., 2015). DPSCs also express basal levels of Nestin, mainly used to evaluate the neurogenic potential of these cells (Young et al., 2016).

Dental pulp stem cells and BMMSCs, the MSCs most used so far, do not show particular morphological or immunological differences (Huang et al., 2009; Isobe et al., 2016), but the extracellular matrix (ECM) that composes their niches is very different. In bone marrow, the fibrillar component consists mainly of type IV collagen, fibronectin and, in a smaller quantity, type I and type III collagen, while the amorphous component is rich in proteoglycan, hyaluronic acid, glycosaminoglycans, etc. In dental pulp type III, collagen is the predominant fibrillar component, followed by type I collagen and amorphous components such as phosphoprotein and glycoprotein (Linde, 1985; Malara et al., 2014).

Decellularized ECM, obtained from bone marrow stem cells, not only preserves the characteristics of *in vivo* ECM but is also biocompatible and promotes cell survival, proliferation, and osteogenic differentiation (Datta et al., 2005; Lai et al., 2010; Xu et al., 2014). Specifically, decellularized bone ECM is able to sustain differentiation of DPSCs toward the osteogenic lineage, determining a significant upregulation of markers involved in osteogenesis without the addition of growth factors, thus making DPSC/bECM (bone ExtraCellular Matrix) hydrogel constructs an interesting biomaterial for bone tissue engineering applications (Paduano et al., 2017). Nowadays, different nanomaterials known to encourage new bone growth (nano-hydroxyapatite, synthetic nano-silicates, and LDHs) have bone therapeutic applications, especially in pathological fractures/injuries such as those derived from osteoporosis (Barry et al., 2016). In addition, some novel biocompatible nanoengineered osteoinductive and elastomeric scaffolds made from biodegradable poly(glycerol sebacate) (PGS) and osteoinductive nanosilicates demonstrated *in vitro* osteogenic differentiation of preosteoblasts and osteogenic properties without persistent inflammation *in vivo* (Kerativitayanan et al., 2017). In this regard, the use of biocompatible material in some oral diseases or during rehabilitation phases after surgery may prevent a rise in oxidative stress levels and ROS production (Tatullo et al., 2012).

Recent studies have shown that use of a chitosan scaffold (Zhang et al., 2016) and the presence of neuronal ECM components such as collagen, laminin, tenascin C, and tenascin R positively contribute to neuronal differentiation of DPSCs (Deng et al., 2014).

The aim of this study was to evaluate the effects of a decellularized ECM, obtained from bone marrow stem cells, on DPSC differentiation toward a neuronal-like phenotype. In detail, we first characterized the matrix, then evaluated both the morphology and the structural organization of DPSC-derived NSCs on ECM, and finally assessed the expression and cellular localization of specific neuronal markers.

## Materials and Methods

### Extracellular Matrix Preparation

Bone marrow-derived mesenchymal stem cells were obtained from healthy donors after they had signed an informed consent form for the treatment of biological material. After expansion in α-MEM with 10% fetal bovine serum (FBS), penicillin, streptomycin, and fungizone added, the cell monolayer was harvested with trypsin/EDTA, and cells were plated at 1–1.5 × 10^4^/cm^2^ on Thermanox or glass slides and cultured for 15 days with α-MEM at 15% FBS. Ascorbic acid (100 μM; Sigma) was added at day 8 after passage, according to Chen et al. (2007), modified by Decaris and Leach (2011). The cells were permeabilized with Triton X-100 containing 15 μl of NH_4_OH in PBS for 7 min at 37°C and then treated with DNAseI (Invitrogen, 100 U/ml PBS) for 1 h at 37°C. After three washes in PBS, the ECM samples were dried for 12 h in a sterile hat. The matrix obtained was stored in the dark at room temperature.

### Dental Pulp Stem Cell Extraction

This study has been reviewed and approved by an Institutional Review Board (IRB) of Azienda Ospedaliero-Universitaria “Policlinico-Vittorio Emanuele” (Catania). The donors/patients are anonymous; their details are known only to the doctor who took the sample and who takes care of their written consent according to the ethical statement. Patients were also informed and agreed to the use of their samples for research purposes. DPSCs were isolated from teeth as described elsewhere (Gronthos et al., 2000; Bonaventura et al., 2018). Briefly, freshly extracted teeth were immediately cracked open, and pulp tissue was collected, minced into small fragments of 1 mm, and then digested in 3 mg/ml collagenase type I (Gibco-Invitrogen, Carlsbad, CA, United States) for 1 h at 37°C. The tissue pellet, after filtration using a 70-μm filter, was resuspended in Dulbecco’s modified Eagle’s Medium containing penicillin G (100 U/ml) and streptomycin (100 μg/ml), which was supplemented with 15% (weight/volume) FBS. Cells, cultured at 37°C in a humidified atmosphere of 5% CO_2_, were subcultured using trypsin/EDTA after reaching 80% confluency. Medium was replaced every 3 days.

### Cytofluorimetric Assay

Cytofluorimetric assay was performed in order to sort a population of CD34^–^ cells coming from dental pulp of freshly extracted teeth as previously reported (Bonaventura et al., 2018). The cells were lightly trypsinized, washed, and resuspended in PBS with 0.1% bovine serum albumin. They were then incubated for 1 h with mouse anti-CD34 primary antibody (Beckman Coulter, Milan, Italy; Item/REF Number: “IM0786”; 0.2 mg/mL) for cell surface marker identification. After three washing procedures, goat anti-mouse IgG FITC-conjugated secondary antibodies were added and incubated for 60 min at room temperature. Three batches of control samples made from human MSCs re-suspended in PBS were incubated only with the secondary antibody. Samples were analyzed using a Coulter Epics XL-MCL flow cytometer (Coulter Corporation, Miami, FL, United States). Control staining with FITC-coupled isotype-matched antibody was performed in a preliminary experiment and never stained >0.3% of CD34^+^ cells. At least 5,000 (forward and side scatter) gated events were collected per specimen. Cells were excited at 488 nm, and the fluorescence was monitored at 525 nm for FITC signal. The fluorescence signals were collected using logarithmic amplification.

### Neuronal Differentiation

The neural differentiation from DPSCs was achieved as previously described (Bonaventura et al., 2015). Briefly, cells were cultured in basic medium and maintained for 6 days in *in vitro* (DIV) culture. The following factors were then added in order to obtain a DMEM differentiation medium: 1 mM dibutyryl cAMP (dbcAMP), 0.5 mM isobutyl methyl xanthine (IBMX), 20 ng/ml human epidermal growth factor (hEGF), 40 ng/ml basic fibroblastic growth factor (bFGF), 10 ng/ml NGF, and 10 ng/ml BDNF. All reagents were purchased from Invitrogen, Milan, Italy. After neural differentiation, cells were cultured for 15 days in a maintaining medium of DMEM added with 10% FBS and 10 μM of retinoic acid. DPSCs differentiated in NSCs were seeded on Thermanox coverslip and microcover glass (24 × 32) with or without ECM at a density of 30,000 cells and cultured for 7 days.

### Scanning Electron Microscopy

The morphological analysis of cells was carried out after adhesion on a rectangular Thermanox coverslip [Electron Microscopy Sciences (EMS), Fort Washington, PA, United States] with or without ECM. Cells were fixed with 2% glutaraldehyde in 0.1 M sodium cacodylate buffer (pH 7.4) for 1 h at 4°C and then post-fixed in 1% osmium tetroxide for 1 h at 4°C. After dehydration in graded ethanol and Critical-Point Drying using CO_2_ (Emitech K850), the coverslips were coated with vacuum-evaporated gold (automatic sputter coater; Agar Scientific) and observed with a Zeiss EVO LS 10 field emission scanning electron microscope.

### Immunofluorescent Assay

Immunofluorescent assay was performed in differentiated DPSCs in order to assess the expression of mature neuronal markers and molecules of adhesion. Briefly, DPSC-derived NSCs were grown on glass coverslips with or without ECM, fixed with 4% paraformaldehyde for 15 min, and then permeabilized with 0.1% Triton X-100 (Sigma–Aldrich) in PBS for 10 min. Mouse anti-NF-H (1:200 dilution; Abcam, Cambridge, United Kingdom; Catalog number: ab187374), mouse anti-MAP-2 (1:300 dilution; Thermo Fisher Scientific, United States; Catalog number: 13-1500), rabbit anti-integrin α-5 (1:1000 dilution; Immunological Sciences, Rome, Italy; Catalog number: ab-10187), and mouse anti-βIII-tubulin (1:1000 dilution; Abcam, Cambridge, United Kingdom; Catalog number: ab78078) primary antibodies were incubated overnight at 4°C. Different secondary antibodies were used, as follows: a FITC-conjugated goat anti-mouse IgG (1:100 dilution; Jackson ImmunoResearch, West Baltimore Pike, PA, United States; Code Number: 115-095-003) was used to reveal NF-H immunoreactivity. A FITC-conjugated goat anti-mouse IgG (1:100 dilution; Santa Cruz Biotecnology, Dallas, TX, United States; Code Number: sc-51614) was used to reveal MAP2 immunoreactivity. An Alexa Fluor 594-conjugated goat anti-rabbit secondary antibody (1:1000 dilution; Immunological Sciences, Rome, Italy) was used to reveal integrin α-5 immunoreactivity. and an Alexa Fluor 594-conjugated rabbit anti-mouse secondary antibody (1:200 dilution; Invitrogen) was used to reveal tubulin immunoreactivity. All secondary antibodies were incubated in a humid chamber for 1 h at room temperature in the dark. Coverslips were washed three times in PBS, mounted with Fluoro Gel with DAPI (Code Number: ab188804; Abcam, Cambridge, United Kingdom) on glass microscope slides. Fluorescence images were then acquired with a CLSM (Zeiss LSM700), using lasers with the appropriate wavelengths (405, 488, and 555 nm), and analyzed using ZEN 2011 software (Federico et al., 2018). The specificity of immunostaining was verified by omitting incubation with the primary or secondary antibody.

### mRNA Isolation

Total mRNA was extracted from the two experimental conditions (DPSC-derived NSCs harvested on tissue culture plates coated or not with ECM) using TRIzol reagent (Thermo Fisher Scientific, Waltham, MA, United States) according to the manufacturer’s instructions. The quality and yield of extracted RNA were evaluated with a NanoDrop 1000 spectrophotometer (Thermo Fisher Scientific, Waltham, MA, United States). Protein fractions were stored at −20°C as phenol–ethanol supernatants awaiting Western blot analysis.

### PCR Amplifications and Semi-Quantitative Analysis

Semi-quantitative RT-PCRs were performed to confirm the expression of neuronal markers (*NF-M*, *NF-L*, *MAP2*) as previously described (Bonaventura et al., 2018). *PAX6* and *VIM* were also measured to assess the differentiation stage. Primer pairs with their relative amplification temperatures (AT) are listed in Table 1. Four micrograms of total RNA was reverse-transcribed with SuperScript^TM^ III Reverse Transcriptase Kit (Thermo Fisher Scientific, Waltham, MA, United States) according to the manufacturer’s instructions. After mRNA conversion, cDNA was used for RT-PCR assays. PCR conditions were set as follows: 1 cycle at 95°C × 2′; 50 cycles at 95°C × 5″, AT × 10″, and 72°C × 5″. Amplicons were run on agarose gel at 2% and visualized by Transilluminator to assess specificity. Densitometric analysis was carried out with ImageJ software^1^ in order to evaluate differences in gene expression levels in semi-quantitative RT-PCR data. Data were normalized to beta-actin as internal control.

**TABLE 1:** List of primer pairs.

**Target**	**Primer F**	**Primer R**	**Expected bp (cDNA)**	**Annealing temperature**
*bACT (NM_001101.5)*	CTTCGCGGGCGACGAT	CACATAGGAATCCTTCTGACCC	103	60
*NF-M (NM_005382.2)*	CTTCCGCTCGCAGTCG	ATTCTGCTGCTCCAGGTAGT	277	55
*NF-L (NM_006158.4)*	TGTGCATGGACCACGCTTAT	TGCTAACCACCGAAGGTTCAA	152	60
*PAX6 (NM_000280.4)*	TCCATCAGTTCCAACGGAGAA	GTGGAATTGGTTGGTAGACAC	337	55
*MAP2 (NM_001363910.1)*	ATTGACAGCCAAAAGTTGAA	TCGAGCAGGTTGATGCTTCC	152	55
*VIM (NM_003380.5)*	CTTCTCTGGCACGTCTTGAC	TCCTGGATCTCTTCATCGTG	94	55

### Western Blot Assay

Proteins were isolated from the previously reserved phenol–ethanol supernatant according to the manufacturer’s instructions. Quantification was performed using NanoDrop Microvolume Spectrophotometers and Fluorometer. In detail, 20 μg of proteins was separated on Bot Gel 4–12% bis tris (Invitrogen), transferred onto a nitrocellulose membrane, and incubated with the primary antibodies at 4°C overnight to reveal MAP2 (1:500 dilution, monoclonal mouse anti-MAP2, Thermofisher), β-III tubulin (1:1000 dilution; Abcam, Cambridge, United Kingdom; Catalog number: ab78078), and β-actin (1:500 dilution; Santa Cruz Biotechnology, Dallas, TX, United States; Code Number: sc-81178). A goat anti-mouse horseradish-peroxidase conjugated secondary antibody was used to reveal the proteins (1:2000 dilution, Santa Cruz Biotechnology, Dallas, TX, United States).

### Statistical Analysis

Data were obtained as mean ± standard deviation. One-way and two-way analysis of variance (ANOVA) were used to assess differences among groups, and statistical significance was assessed by the Tukey–Kramer *post hoc* test. For Western blot and immunofluorescence analysis, a *t*-test was used to assess differences between the two experimental groups. The level of significance for all statistical tests was *p* < 0.001. All statistical analyses were performed using GraphPad Prism 7.

## Results

### Morphological Appearance of Extracellular Matrix

**FIGURE 1:**
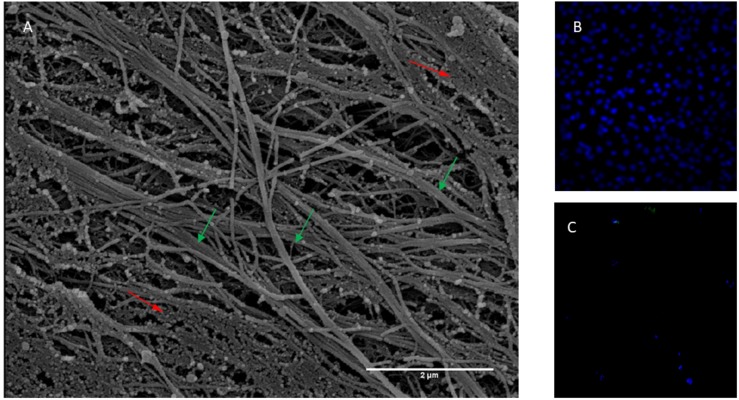
**(A)** Scanning electron micrograph of ECM obtained from bone marrow stem cells. Banded fibres are indicated with green arrows while body cellular residues with red arrows. Thickness of fibres 100 nm about. Magnification 20,000×. Scale Bar 2 μm. **(B)** Not decellularized and **(C)** decellularized extracellular matrix stained with DAPI.

In the present study, mesenchymal cells from healthy donor bone marrow were used to fabricate an ECM bioscaffold in order to test its influence on neuronal differentiation. The ECM, decellularized with previously described procedures (Chen et al., 2007; Decaris and Leach, 2011), appeared as a fibrillar stroma composed of thick bundles following the orientation of cellular bodies irregularly intersected with thin filaments. The network was constituted by fibers and filaments of various sizes, among which collagen fibers were particularly identifiable due to both their banded structure and their size when observed at higher magnification (Figure 1A). The permeabilization and DNase method used for the free cell ECM preparation did not allow total removal of cellular material. However, DAPI staining shows that decellularized ECM does not show nuclei compared with samples before decellularization (Figures 1B,C). Very thin, slightly banded filaments were present inside these cytoplasmatic residues.

### Morphology and Ultrastructural Organization of the DPSC-Derived Neuronal-Like Stem Cells Cultured on ECM

**FIGURE 2:**
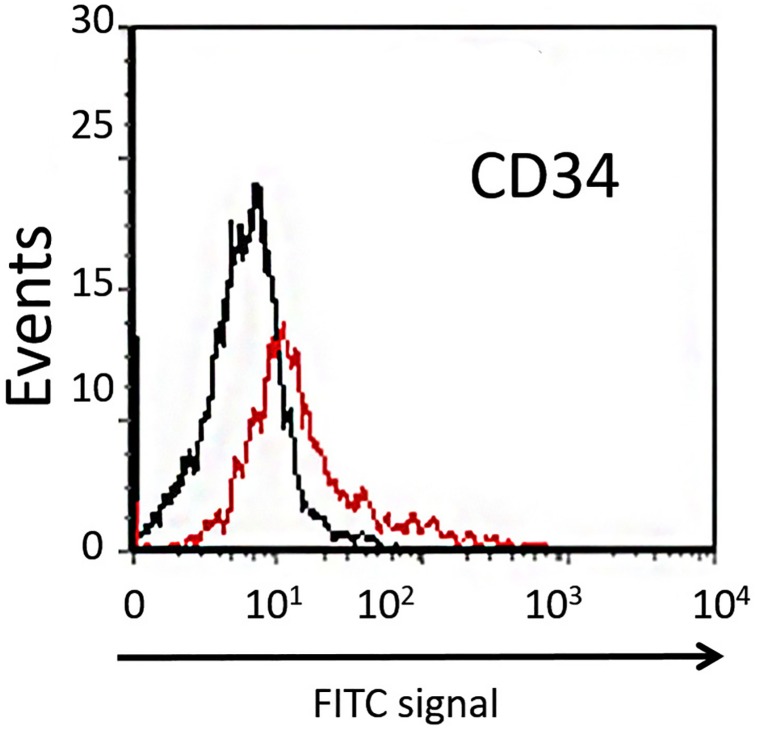
Cytofluorimetric analysis of DPSCs. Black line is the sub-population of CD34^–^ cells labeled with FITC-coupled isotype-matched antibody. Red line is the sub-population of FITC-labeled CD34^+^ cells.

Dental pulp stem cells can be divided into two different subpopulations based on their expression of CD34. The majority of DPSCs (about 98%) are CD34^–^, showing higher clonogenic potential and plasticity compared with the CD34^+^ fraction (Stevens et al., 2008). In order to isolate the CD34^–^ subpopulation of DPSCs extracted from dental pulp, the cells were sorted using flow cytofluorimetric assay. In our study, the mean percentage of CD34^–^ cells sorted was about 90% (Figure 2), and these were used in further experiments.

To examine the effect of the ECM on neuronal differentiation and structural organization, we observed the morphology of cells cultured on both the Thermanox substrate and the ECM by SEM microscopy.

**FIGURE 3:**
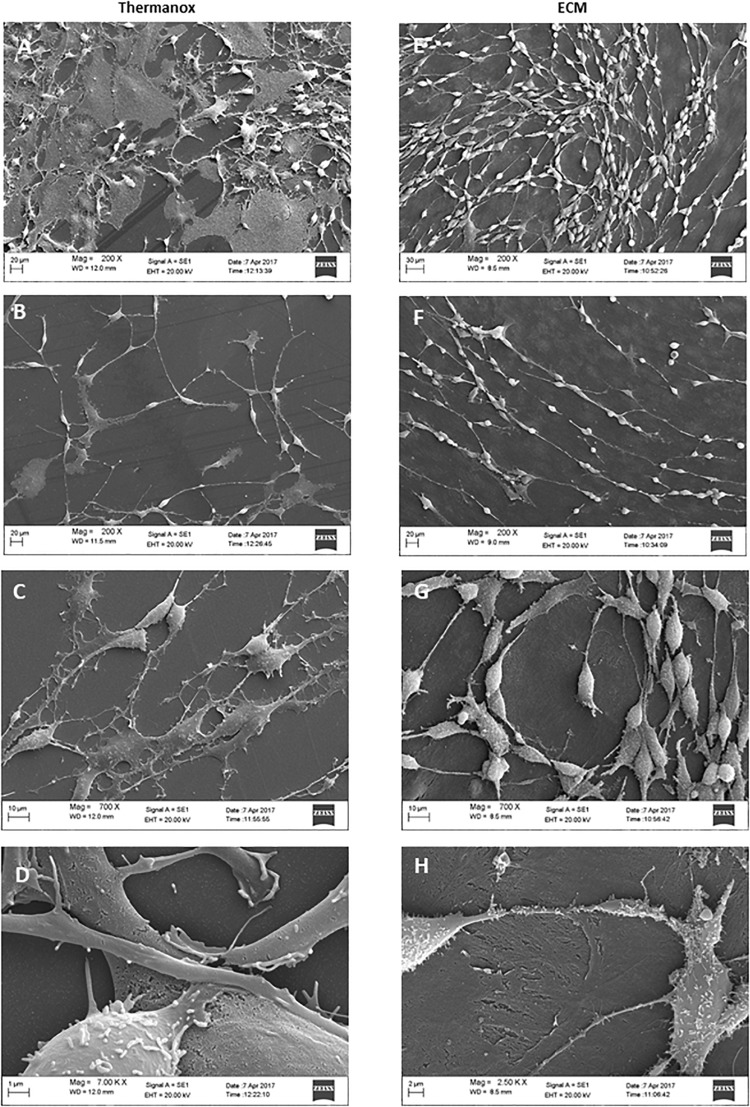
Electron micrographs of DPSC-derived neuronal-like stem cells grown for 7 days on Thermanox **(A–D)** and on ECM **(E–H)**. Thermanox samples showed a polygonal morphology and filipodia (neurites) that follow a random orientation, unlike cells grown on ECM, which show a fusiform structure and long filipodia that follow matrix fibers. Scale bars: **(A,E,D)** 20 μm; **(B,F)** 10 μm; **(C)** 1 μm; **(G)** 2 μm; **(H)** 30 μm.

When grown on Thermanox support, DPSC-derived NSCs showed a random arrangement and did not exhibit a specific orientation (Figure 3A). Cytoplasmic projections connected with each other to constitute an irregular mesh (Figure 3B). The plasma membrane had an apparently homogeneous surface with a small number of microvilli (Figure 3C). The majority of cells exhibited a polygonal, flattened morphology with a wide cytoplasmic area lying on the substrate (Figure 3D).

In contrast, DPSC-derived NSCs grown on the ECM exhibited more peculiar structural features: cells visibly followed the orientation given by the fibers of the matrix (Figure 3E) and formed a network. Single cells showed a fusiform structure with long projections (Figure 3F), and cell surfaces were variously configured: more or less long filipodia and numerous microvilli-like structures (Figure 3G). Very few cells lay with a polygonal shape on the ECM (Figure 3H).

### Effect of ECM on Cell Adhesion and Neuronal Markers

**FIGURE 4:**
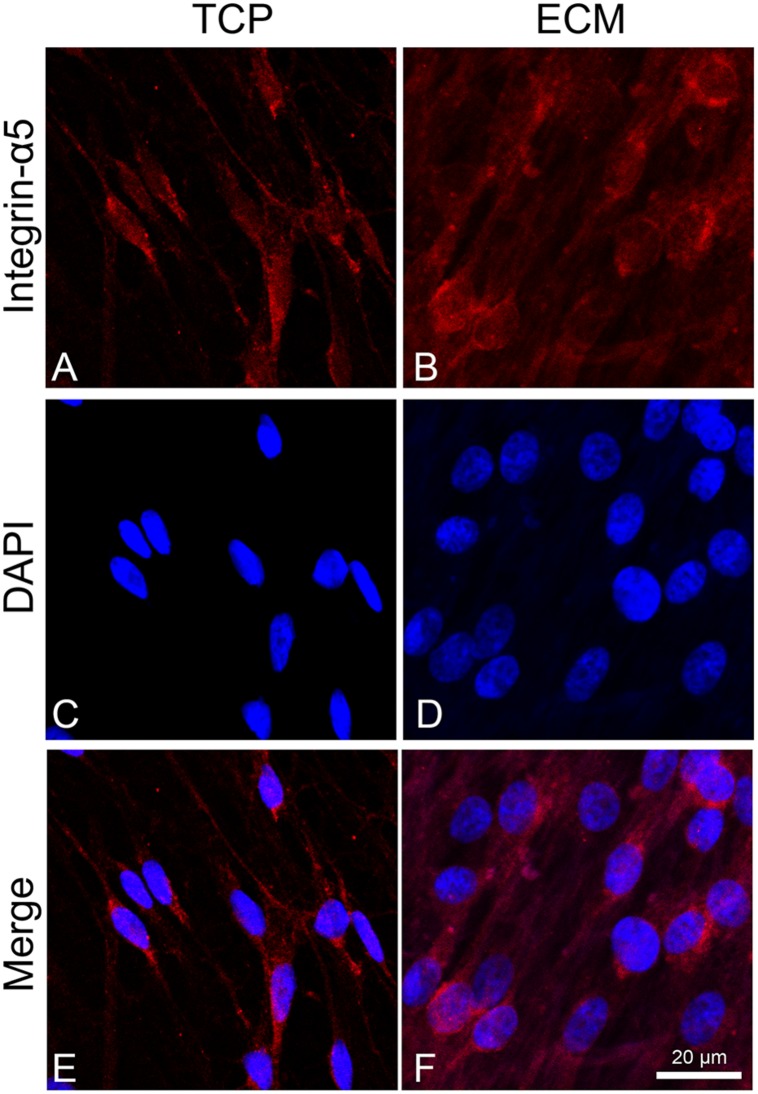
Distribution of the integrin α-5 (red signals) in DPSC-derived neuronal-like stem cells grew for 7 days on glass **(A)** and ECM **(B)** support. Nuclei were stained with DAPI **(C–D)** and a merge was made **(E–F)**. Images were captured using lasers with wavelenght of 405 and 555 nm. Scale bar corresponds to 20 μm.

To assess the adhesion process of cells on the two different substrates (glass or ECM), we observed the distribution of integrin α5 by immunofluorescence. This integrin is known to join with the β1 subunit to form the primary fibronectin receptor α5β1 and, in the central nervous system, is involved in the process of neuronal migration and in long-term potentiation (LTP). Inhibition or deletion of integrins, such as α3, α5, or β1, negatively affects synapse organization and impairs LTP (Dityatev et al., 2010; Marchetti et al., 2010; Lilja and Ivaska, 2018). As shown in Figure 4, integrin α5 immunoreactivity in cells cultured on glass had a cytoplasmatic localization near the perinuclear zone, while, in ECM-cultured cells (Figure 4B), its immunoreactivity appeared more distinctly expressed across the cell adhesion surface, particularly localized at the level of cytoplasmic projections.

**FIGURE 5:**
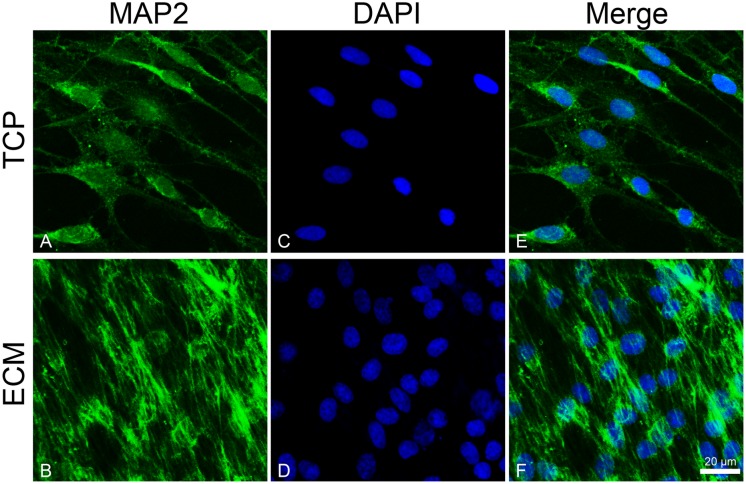
Distribution of the MAP2 (green signals) in DPSC-derived neuronal-like stem cells grew for 7 days on glass **(A)** and ECM **(B)** support. MAP2 was detected by a mouse primary antibody followed by a FITC-conjugated goat anti-mouse secondary antibody. Nuclei were stained with DAPI **(C–D)** and a merge was made **(E–F)**. Images were captured using lasers with wavelenght of 405 and 488 nm. Scale bar corresponds to 20 μm.

**FIGURE 6:**
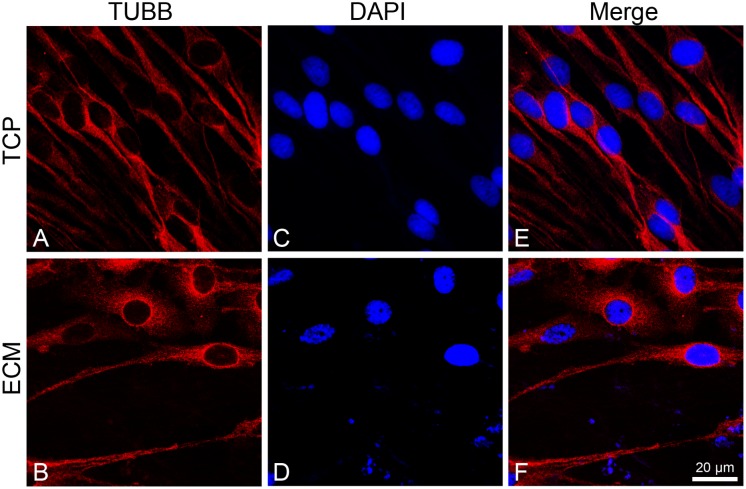
Distribution of βIII-tubulin (red signals) in DPSC-derived neuronal-like stem cells grew for 7 days on glass **(A)** and ECM **(B)** support. Nuclei were stained with DAPI **(C–D)** and a merge was made **(E–F)**. Images were captured using lasers with wavelenght of 405 and 555 nm. Scale bar corresponds to 20 μm.

To investigate the effect of the substrates on the induction of DPSCs toward the neuronal differentiation lineage, we performed immunofluorescence experiments. Immunofluorescence reactions were carried out to evidence MAP2, β-III tubulin, and neurofilament-heavy (NF-H) markers. As shown in Figures 5, 6, both glass-cultured and ECM-cultured cell lines showed immunoreactivity for MAP2 and β-III tubulin. In the glass-cultured cells, MAP2 is located in the cytoplasm, with a large number of signals in the main cell body. In the ECM-cultured cells, MAP2 is largely present in the cytoplasm, either in the main cell body or in the cytoplasmatic extensions (filopodia). In the glass-cultured cells, as well as in the ECM ones, βIII-tubulin is located in the cytoplasm, including the filopodial regions.

**FIGURE 7:**
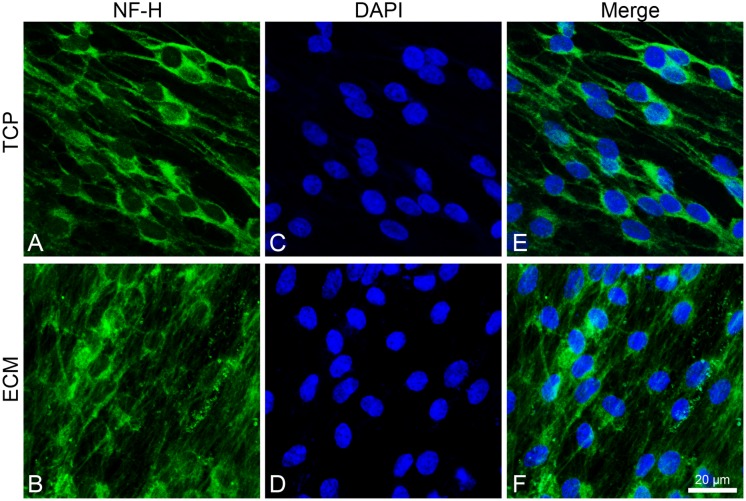
Distribution of NF-H (green signals) in DPSC-derived neuronal-like stem cells after 7 days of culture. In DPSC-derived neuronal-like stem cells grew on glass **(A)** NF-H is widely observed in central area; on ECM **(B)** more cells exhibit polymerized neurofilament along neurites. Nuclei were stained with DAPI **(C–D)** and a merge was made **(E–F)**. Images were captured using lasers with wavelenght of 405 and 488 nm. Scale bar corresponds to 20 μm.

Both experimental groups also showed immunoreactivity for NF-H, with the same differences (Figure 7). In the glass group, cells show an NF-H distribution in the perinuclear area; in ECM-cultured conditions, they exhibited polymerized NFs along filopodia.

### Expression Levels of Mature Neuronal Markers

**FIGURE 8:**
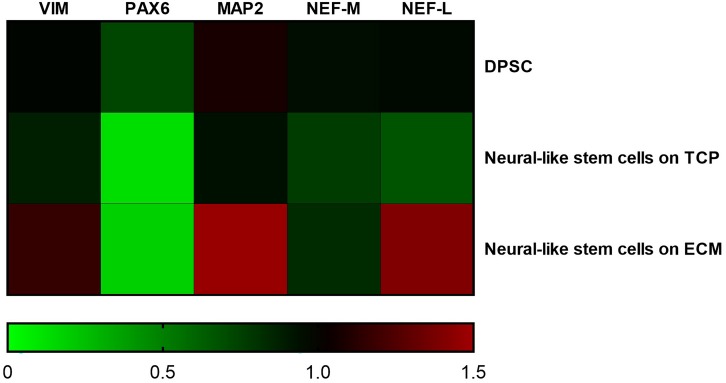
Gene expression change during differentiation. Densitometric results of semi-quantitative RT-PCR analysis demonstrate a significant increase in early (PAX6 and VIM) and mature (MAP2, NF-L, and NF-M) neuronal markers. mRNA levels were normalized to the amount of β-actin mRNA and represented in a heatmap. The data are expressed as mean ± standard deviation (***P < 0.001 vs. Control as determined by two-way ANOVA followed by Tukey–Kramer *post hoc* test) from at least three independent experiments.

**FIGURE 9:**
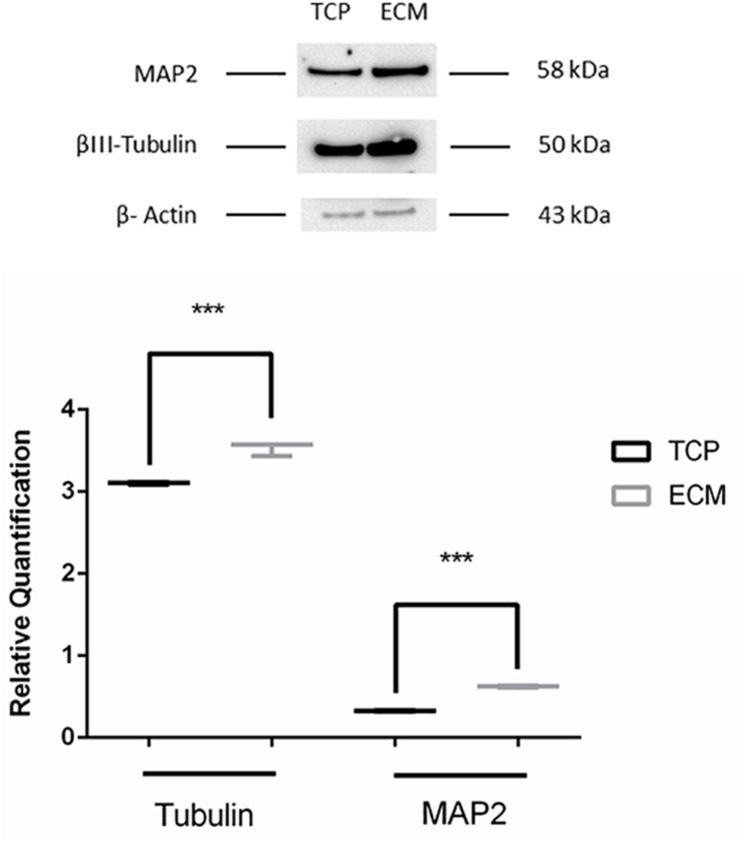
Western blot assay. The data are expressed as mean ± standard deviation (***P < 0.001 vs. Control as determined by Student’s *t*-test) from at least three independent experiments.

Supportive findings came also from RT-PCR experiments, as shown in Figure 8, and from Western blot, as shown in Figure 9. We decided to measure the differential expression of a set of genes, including two markers of the differentiation state (*PAX6* and *VIM*), as well as four mature neuronal markers (*MAP2*, *NF-L*, *NF-M*, and *βIII-tubulin*). Results from semi-quantitative RT-PCR assays showed a statistically significant increase in the *Vimentin* (^∗∗∗^*P* < 0.001), *MAP2* (^∗∗∗^*P* < 0.001), and *NF-L* (^∗∗∗^*P* < 0.001) expression level in cells cultured on ECM compared to non-coated TCP. *PAX6* (Paired box 6), which is known to be expressed only in the early phases of differentiation, decreased progressively during the differentiation protocol in both cell groups compared to controls (^∗∗∗^*P* < 0.001). Contrary to what was expected, NF-M expression levels decreased in both cell groups compared to controls (^∗∗∗^*P* < 0.001). Results from Western blot showed a statistically significant increase in the *MAP2* (^∗∗∗^*P* < 0.001) and *βIII-tubulin* (^∗∗∗^*P* < 0.001) of cells grown on ECM compared with samples grown on TCP.

## Discussion

To date, neurodegenerative diseases and central nervous system injuries are the main cause of invalidity. There is therefore an urgent need to develop a valid experimental *in vitro* cell model to study neuronal damage. As previously observed (Isobe et al., 2016), MSCs (i.e., bone marrow MSCs and umbilical cord stem cells) do not all have the same propensity to become neuronal stem cells. Among these, DPSCs seem to be the easiest to induce toward the neuronal differentiation lineage because of their neuroectodermal origin.

Usually, during *in vitro* experimentals, cells differentiation occurs in contact with plastic or glass. However, in physiological conditions, stem cells are located within a microenvironment (the *cellular niche*) that influences cell proliferation and specialization (Scadden, 2006). A number of studies previously conducted on the osteogenic and chondrogenic properties of MSC have shown that the presence of a bio-scaffold can positively contribute to cell differentiation (Datta et al., 2005; Lai et al., 2010; Cai et al., 2015; Xing et al., 2015; Tang et al., 2016). Similarly, it has been observed that the presence of a chitosan scaffold (Zhang et al., 2016) and the use of a biogel containing an ECM component (Deng et al., 2014) could promote NSC differentiation.

Based on these data, in this study, we evaluated the effects of a decellularized ECM scaffold on the neuronal differentiation of CD34^–^ DPSCs. Morphological data from scanning electron microscopy show that the ECM scaffold induced cells to acquire a neuronal-like organization, with a central body and long cytoplasmic extensions that follow the underlying fibers, which work as “rails.” Moreover, ECM seems to influence the adhesion to the substrate, as shown by the immunoreactive signals of the α-5 integrin, which is known to be implicated in dendritic development and neuronal migration (Dityatev et al., 2010; Marchetti et al., 2010).

In addition, ECM is able to influence the expression and distribution of a set of mature neuronal markers (*MAP2*, *NF-L*, *NF-H*, and *VIM*) and differentiation markers (*PAX6*), as demonstrated by immunofluorescence and RT-PCR assays. MAP2 is expressed from the early stage of neuronal differentiation. At the beginning, it is localized in neurites, while in mature neurons, MAP2 is seen to be localized in dendrites (Mohan and John, 2015). Under our experimental conditions, MAP2 shows a greater immunoreactivity along neurites in ECM-cultured cells than in glass-cultured cells, as also confirmed by the increased mRNA and protein expression. NFs are intermediate filaments whose expression increases during neuronal differentiation. They are finally assembled in a heteropolymer consisting of three subunits (NF-L, NF-M, and NF-H) whose function is to protect neurons from mechanical stress and ensure cell elasticity (Gentil et al., 2015; Lowery et al., 2015). Vimentin is another intermediate filament expressed during neuronal differentiation and is involved in stretching of neuritis, whose expression is usually arrested with the stop of the mitotic cycle (Cochard and Paulin, 1984). It has been observed that vimentin and NF co-exist within the same neurite, forming heteropolymers, even if at very low percentages (Yabe et al., 2003), which contribute to NF deposition (Sun et al., 1997). Our results show that NF-H is more diffused along cytoplasmic extension on the ECM-cultured cells than on those grown on glass. Similarly, RT-PCR data show that the ECM-cultured DPSC-derived NSCs express a greater amount of VIM and NF-L, probably due to the presence of matrix collagenous fibers that promote neurite elongation.

Finally, we measured the expression of *PAX6*, a transcription factor active mainly in the early stage of neuronal differentiation and later replaced by other transcription factors (Zhang et al., 2010). As revealed by semi-quantitative RT-PCR data, ECM-cultured DPSC-derived NSCs express a statistically significant higher level of PAX6 compared to TCP-cultured cells (^∗∗^*P* < 0.005). These data are in line with other results reported by Hamamoto et al. (2011) who showed that pancreatic β-cells had a higher expression of PAX6 when they grew on ECM due to interaction between integrins and ECM components; at this differentiation state, ECM seemed not to reduce the number of the few PAX6 + neuroprogenitors (Basuodan et al., 2018).

## Conclusion

Dental pulp stem cells appear, on the grounds of their proliferative capacity and their propensity to differentiate into NSCs, to be good candidates for developing a valid *in vitro* model of human neuronal lineage. Moreover, since their propensity for differentiation was increased by the presence of the ECM, this latter could be used as support for the growth and differentiation of mesenchymal cells. However, a number of limitations to this study remain. First, the isolation of DPSCs or other dental stem cells, of course, requires a dental extraction; therefore, preventive conservation and maintenance protocols of previously isolated cells should be developed to make them available for patients when necessary. Secondary, BMMC isolation is not so easy to perform. It would be appropriate to study the effect of ECM derived from other sources of mesenchymal cells taken from anatomical compartments that are easier to access, such as skin fibroblasts. Finally, we investigated the role of ECM in enhancing the neuronal induction of DPSCs, but we did not address the differentiation of specific neuronal phenotypes. Further studies are needed to clarify the role of ECM in the maturation of DPSC-derived NSCs toward particular neuronal subpopulations by measuring specific differentiation markers or electrophysiological activity in order to create more accurate models to be applied in future for neurodegenerative conditions or neuro-regeneration studies.

## Data Availability Statement

The datasets generated for this study are available on request to the corresponding author.

## Ethics Statement

This study was reviewed and approved by the Institutional Review Board (IRB) of Azienda Ospedaliero-Universitaria “Policlinico-Vittorio Emanuele” (Catania). The donors/patients are anonymous; their details are known only to the medical doctor who took the sample and who takes care of their written consent following the ethical statement. All subjects gave written informed consent in accordance with the Declaration of Helsinki.

## Author Contributions

GB and FS conceived and coordinated the study. SL performed all experiments. RI and VL performed and analyzed the PCR experiments. SS performed the immunofluorescent image acquisition and GV prepared the ECM. MB and SC performed a critical revision of the manuscript. All authors reviewed the results and contributed to writing of the manuscript.

## Conflict of Interest

The authors declare that the research was conducted in the absence of any commercial or financial relationships that could be construed as a potential conflict of interest.

## Footnotes

1 https://imagej.nih.gov/ij/
